# Neural substrates of L2-L1 transfer effects on phonological awareness in young Chinese-English bilingual children

**DOI:** 10.1016/j.neuroimage.2024.120592

**Published:** 2024-03-26

**Authors:** Jia-Wei Kou, Li-Ying Fan, Hsin-Chin Chen, Shiou-Yuan Chen, Xiaosu Hu, Kehui Zhang, Ioulia Kovelman, Tai-Li Chou

**Affiliations:** a Department of Psychology, National Taiwan University, Taipei, Taiwan; b Department of Education, National Taipei University of Education, Taipei, Taiwan; c Department of Psychology, National Chung Cheng University, Chiayi, Taiwan; d Department of Early Childhood Education, University of Taipei, Taipei, Taiwan; e Department of Psychology, University of Michigan, Ann Arbor, MI, USA

**Keywords:** Bilingualism, Learning, Development, Phonology, fNIRS

## Abstract

The growing trend of bilingual education between Chinese and English has contributed to a rise in the number of early bilingual children, who were exposed to L2 prior to formal language instruction of L1. The L2-L1 transfer effect in an L1-dominant environment has been well established. However, the threshold of L2 proficiency at which such transfer manifests remains unclear. This study investigated the behavioral and neural processes involved when manipulating phonemes in an auditory phonological task to uncover the transfer effect in young bilingual children. Sixty-two first graders from elementary schools in Taiwan were recruited in this study (29 Chinese monolinguals, 33 Chinese-English bilinguals). The brain activity was measured using fNIRS (functional near-infrared spectroscopy). Bilingual children showed right lateralization to process Chinese and left lateralization to process English, which supports more on the accommodation effect within the framework of the assimilation-accommodation hypothesis. Also, compared to monolinguals, bilingual children showed more bilateral frontal activation in Chinese, potentially reflecting a mixed influence from L2-L1 transfer effects and increased cognitive load of bilingual exposure. These results elucidate the developmental adjustments in the neural substrates associated with early bilingual exposure in phonological processing, offering valuable insights into the bilingual learning process.

## 1. Introduction

### 1.1. Bilingualism: cross-linguistic difference leads to transfer effect

Bilingual education has emerged as a growing trend, fueled by the increasing demand for English proficiency in today’s globalized world. As such, many schools outside English-speaking areas have implemented bilingual programs to provide students with opportunities to develop their English language and literacy skills. On the one hand, bilingual research suggests that cross-linguistic transfer mechanisms should reciprocally support children’s emerging dual-language literacy skills. On the other hand, there are substantial cross-linguistic distances for example between English and Chinese, with differences spanning both spoken and orthographic word structures. Our inquiry takes a neuroimaging approach to uncover the effects of bilingual interactions on children’s phonological awareness literacy skill in the context of bilingual Chinese-English elementary school education in Taiwan.

#### 1.1.1. Theoretical perspectives on word reading

Theoretical perspectives on word reading generally posit that children’s emerging literacy builds upon the strengthening of their sound, meaning, and orthographic literacy skills and the growing associations between them. Cross-linguistic differences may influence children’s emerging sound-to-print and meaning-to-print interconnections (Zhang et al., 2023). These theoretical assumptions are well supported by research evidence that had, for instance, compared monolingual English speakers in the US and monolingual Chinese speakers in China, demonstrating that phonological awareness plays an important role both in Chinese and English acquisition. Yet, phonological literacy skills take precedence in the beginning readers of English, whereas morphological skills take precedence in the beginning readers of Chinese (grade 2 comparison in McBride-Chang et al., 2005, Ruan et al., 2018). In sum, there are cross-linguistic differences in the relative role that phonological awareness plays in learning to read in English versus Chinese, especially in beginning readers.

Importantly, in the case of bilingual learners, the two languages are thought to interact and thus lend reciprocal support for children’s emerging dual-language literacy skills. According to the interactive transfer framework (ITF, Chung et al., 2019), cross-linguistic transfer is governed by a set of core factors that include cross-linguistic generalizability of a given literacy skill, cross-linguistic distances, and children’s proficiency in each of their languages. Among all the different literacy skills, phonological awareness literacy skill is considered to be one of the easy-to-transfer skills as the ability to, for instance, segment words into syllables and sounds, is relatively generic across languages. Even though phonological awareness is not the most influential literacy skill in Chinese, the learning path could be modified by bilingual experiences. Bilingualism is one approach to uncovering developmental plasticity in learning to read, offering a lens to understand better how learning experiences interact with children’s emerging neural architecture for foundational literacy skills such as phonological awareness.

Comparisons between bilingual and monolingual learners also suggest positive cross-linguistic influences of bilingualism on children’s phonological literacy skills. In particular, the ITF perspective posits that literacy features that are more salient or more predictable in one of the bilinguals’ languages, may have a positive transfer effect on the language where these features are more subtle or complex. In support of this theoretical perspective, Chen et al. (2010) have found that following intense English language instruction, bilingual Mandarin-L1 primary school students in China showed stronger phonological awareness skills in Chinese relative to their monolingual Chinese counterparts. Nevertheless, this bilingual facilitation effect was observed only after two years of English literacy instruction suggesting that some threshold of English L2 proficiency must be reached before the transfer effects became noticeable in the children’s dominant language. Some studies have observed the emergence of positive transfer effects earlier (e.g., 1st grade) in the course of bilingual instruction, but these studies were more likely to include children with earlier (e.g., pre-school) bilingual exposure, bilingual environment (e.g., U.S., Singapore), and/or more intense L2 instruction (e.g., immigration/immersion; Hsu et al., 2019; Sun et al., 2021; Yeong and Rickard Liow, 2012).

The present study seeks to understand the nature of cross-linguistic transfer of phonological awareness literacy skills, a critical stepping stone to proficient reading, in beginning readers. Prior bilingual work suggests robust phonological awareness transfer even for such distant language pairs as English and Chinese (Chung et al., 2019). Nevertheless, the relation between years of English exposure and levels of proficiency before such transfer becomes noticeable remains unclear. To shed light on this unknown course, we examine 1st-grade bilingual children with pre-school exposure to both English and Chinese, in a Chinese-dominant language environment in Taiwan. Compared to the previous study of Chen et al. (2010), English was more common in Taiwan nowadays, and the first graders in the current study already had at least 1 year of pre-school English learning experience. The L2-richer environment and English exposure in earlier developmental stages may lead to the emergence of positive transfer effects, which is supported by a previous study (Yeong and Rickard Liow, 2012). Importantly, neuroimaging studies can potentially reveal group differences in phonological processing even when participant groups are matched for their proficiency. These neuroimaging effects are particularly noticeable in studies of dyslexia (when readers are matched for proficiency but differ in age as in Kovelman et al. (2012)), but have also been found in young bilinguals (Zhang et al., 2023). We thus took a neurocognitive approach to our bilingual inquiry.

### 1.2. Brain bases of bilingual language processing

Most of our knowledge of the neural bases of reading and its development stems from research with alphabetic languages (Mathur et al., 2020; Wagley and Booth, 2022; Weiss et al., 2018; Yan et al., 2021). In these studies, the phonological neural networks (the dorsal pathway; Hickok and Poeppel, 2007) have been reported as a pivotal role in the acquisition of phonological processing skills (Yu et al., 2018), and the acquisition of word reading, in line with prior behavioral results (Wagley and Booth, 2022). In cross-sectional developmental research using English-speaking and Chinese-speaking participants, various regions in the phonological networks, especially the left superior temporal gyrus (STG), were more activated in the comparison between English-speaking children and adults during phonological judgments. In contrast, only the left dorsal inferior frontal gyrus (dIFG) increment was observed in the Chinese-speaking participants (Brennan et al., 2013). In sum, cross-linguistic comparisons suggest developmental differences in how the dorsal neural pathways function during phonological awareness literacy tasks in English and Chinese.

For young Chinese-speaking children, relatively limited research has examined the neural basis of Chinese phonological processing. Yet as in the Brennan et al. (2013)’s results, the importance of the left frontal regions (especially the IFG) in Chinese phonological processing was highlighted in adult studies (Li et al., 2022; Zhu et al., 2014), beginning learners of L2 Chinese (Deng et al., 2011), and dyslexia children (Yan et al., 2021; Zhang et al., 2021). This finding might result from the opaqueness of Chinese, which leads to additional cognitive demand on the manipulation of phonemic units during phonological judgments (Cao et al., 2011; Kita et al., 2013). However, as one of the few studies that focuses on Chinese-speaking children, Cao et al. (2011) found increasing activation in the left STG between the ages of 9 and 11. Cao et al. (2011) finding different from Brennan et al.’s study (2013) might be because of the age of their participants. Cao et al. (2011) recruited participants from age 8 to 12, without further dividing them into smaller age groups. Therefore, it is still unclear during the preschool phase, whether the left STG or IFG, or both, would be crucial to Chinese phonological development, especially in the premise of bilingual exposure to English L2.

The neural mechanisms of bilingual language processing have been a widely-studied topic. L2 processing was found to activate brain networks similar to those activated in L1 processing in some earlier studies (Illes et al., 1999; Nakada et al., 2001). But many other studies have found additional brain involvement in L2 processing, suggesting the L1-L2 transfer effect (Durgunoǧlu et al., 1993; Hsu et al., 2019; Verbeek et al., 2022). This discrepancy could be understood in the framework of assimilation-accommodation hypothesis, which suggests that bilinguals may use either assimilation or accommodation strategies from L1 in L2 processing, leading to different neural mechanism in L2 processing (Cao, 2016; Nelson et al., 2008; Perfetti and Liu, 2005).

Besides the influence from L1 to L2, recent bilingual research also found that, despite the fact that L1 is the dominant language developed in early life, changes in the brain networks involved in L1 processing still may occur (Bice and Kroll, 2015; Brice et al., 2021; Kroll and Mendoza, 2022; Otwinowska et al., 2021). On the other hand, bilinguals also may allocate more cognitive resources to learn two languages simultaneously (Folke et al., 2016; Jones et al., 2012). Therefore, if the development of phonological awareness has not yet benefited from bilingual exposure, then the cognitive load of abundant dual-language information would possibly result in more support or compensation from outside the dorsal neural pathways or even the right hemisphere (Cao et al., 2011; Goto et al., 2015).

A recent bilingual study also examining Chinese-English bilingual children could be a reference (Zhang et al., 2023). In Zhang et al. (2023) bilingual children, the heritage language was Chinese with relatively low Chinese proficiency. Their bilingual participants were in an English-dominant environment. Compared to the English monolingual children, the language proficiency and behavioral differences of bilingual children were not profound. Yet, these two groups of children showed significant differences in brain activation. In both the PA (phonological awareness) and MA (morphological awareness) tasks, their bilingual children showed more right brain activation relative to monolinguals, which was attributed to a supportive role on the attention demands for dual language use. Also, in the PA task, bilingual children showed less activation in the left IFG, indicating benefit from the dual-language exposure. In contrast, in the MA task, bilingual children showed more activation in the left IFG, which may reflect the cognitive load on the language-specific morphology computations.

These aforementioned results extend the current understanding of neural substrates of Chinese phonological processing in Chinese-English bilingual children. To further explore this English-to-Chinese transfer effect, we would additionally need a Chinese monolingual group for a comparison group. Also, it would be crucial to recruit bilingual children with relatively high Chinese proficiency as compared to bilingual children with relatively low Chinese proficiency in Zhang et al. (2023) study. Our bilingual group would allow us to elucidate a more Chinese influence on phonological transfer. Therefore, the present study takes these factors into account to examine L2-L1 transfer effects on phonological awareness.

### 1.3. The present study

We aimed at uncovering the effects of preschool second language experience on children’s phonological literacy skills during the earliest periods of reading development. To address this aim, we employed a neuro-cognitive approach of comparing Chinese–speaking children growing up in Taiwan, with one group experiencing a standard monolingual Chinese upbringing and another group that had at least one year of English learning experience prior to enrolling in a bilingual Chinese-English education program.

Chen et al. (2010) recruited first graders in an environment with a “lack of exposure to authentic English” before formal English education. Their third graders were found to have an L2-L1 transfer effect after receiving two years of formal English education, which is different from the current study. In the current study, English was more common in the environment, and our first graders already had at least 1 year of English learning experience. Therefore, the current study aims to try to examine the L2-L1 transfer effect in an L2-richer environment and at an earlier developmental stage. Another line of evidence recruiting bilingual children with at least 1 year of English exposure is that the transfer effect from L2-English to L1-Chinese is observable in preschool sessions in a bilingual environment. For example, kindergartens in Singapore have developed Chinese phoneme awareness after kindergarten exposure to more oral and written English (Yeong and Rickard Liow, 2012).

Grade 1 (age 6–7) children completed the auditory phonological task during fNIRS neuroimaging and behavioral literacy assessments in each language. For the Chinese language in bilinguals, we predicted a positive transfer effect from English, which has more salient and transparent sound-to-print characteristics than Chinese. In particular, we predicted that the bilinguals might show stronger engagement of the regions of the dorsal neural networks (e.g. the left STG and IFG), reflecting the increased sensitivity to phonological information compared to Chinese monolinguals. We further predicted that as phonemes in English words may be easier to segment, the phonological information as well as orthographic and semantic information would be faster to be extracted in English processing. This may lead to stronger and more widespread engagement of core language regions (including the IFG, STG/MTG, and parietal regions) in bilinguals during English processing than Chinese processing. Understanding the neurocognitive bases of emerging bilingual literacy is important in the context of growing global interest in dual-language programs where children are learning to speak and read in a language that differs from the language of their community. The present study aims to illuminate mechanisms guiding the dual-language development of phonological literacy skills, a critical stepping stone to reading, to inform both bilingualism perspectives as well as literacy theories aiming to explain reading development across languages and across learners.

## 2. Methods

### 2.1. Participants

Sixty-two first graders (age 6–7) from elementary schools in Taipei were recruited in the present study, including 29 Chinese monolingual children (48 % male, *M*_*age*_= 7.00, *SD* = 0.42) and 33 Chinese-English bilingual children (43 % male, *M*_*age*_= 6.85, *SD* = 0.20). All parents completed a modified version of a previously validated and published Bilingual Language Background and Use Questionnaire (Kovelman et al., 2008). The questionnaire comprised items pertaining to children’s cognitive and language development, history of physical health, home and school language background. All participants in the bilingual group had learned English before the age of 5, with a minimum of 1 year of English exposure prior to testing, while all participants in the monolingual group had less than 1 year of English learning experience, which is substantially lower than the more consistent prior and concurrent exposure to English in the bilingual group. For the children’s language background, 83 % of the parents in the monolingual group agreed with the item “Does your child speak only Mandarin Chinese 100 % of the time?”, while only 3 % of the parents in the bilingual group agreed with that. All participants were neurotypical children who were native speakers of Mandarin Chinese, with normal hearing and normal or corrected-to-normal vision. Most participants were right-handed (R: 60, L: 2). Informed consent and parental consent were obtained from all child participants and their parents after providing them with comprehensive study information. This study was reviewed and approved by the Research Ethics Committee of National Taiwan University.

### 2.2. Behavioral assessments

All child participants completed standardized or experimenter-made Chinese language and literacy tasks, assessing the following literacy or cognitive skills. The performance can be found in Table 1.

#### 2.2.1. Chinese phonological awareness

Chinese phonological awareness was assessed using a Chinese version of the Elision subset of the comprehensive test of phonological processing (CTOPP) based on Newman et al. (2011). Participants were asked to delete a part of the word and answer the remaining part of the word, including 36 items with 6 elisions on the syllable level and 30 of them on the phoneme level. (e.g., Can you say “/fuˊ/”? Can you say “/fuˊ/” without the “/f/” sound? 請跟我念“福 /fuˊ/”, “福 /fuˊ/” 去掉 “/f/”是什麼呢? – 無 /uˊ/).

#### 2.2.2. Chinese morphological awareness

Chinese morphological awareness was assessed based on Song et al. (2015). Participants were given the rule of some compound words and were asked to construct a new compound word with a new morpheme based on the given rule (e.g., Here is a chain on the hands, we call it a hand chain; How do we call a chain on the feet? – a foot chain. 這裡有條 鏈, 我們把它帶在手上面, 我們叫它手鏈. 這裡有條鏈, 我們把它帶在腳上面, 我們叫它? – 腳鏈).

#### 2.2.3. Chinese receptive vocabulary

Chinese receptive vocabulary was assessed using the Peabody Picture Vocabulary Test-Revised (PPVT-R; Lu and Liu, 1998).

#### 2.2.4. Chinese character recognition and reading

To assess the ability to recognize and read Chinese characters, participants were asked to choose the character that was read to them from four characters they saw in each trial in the recognition task (30 items), and read out another set of characters in the reading task (60 items).

#### 2.2.5. Chinese sentence reading fluency

The Chinese sentence reading fluency assessment was modified based on the Reading Fluency subtest of Woodcock-Johnson IV (WJ-IV; Schrank et al., 2014). Children were asked to read 100 Chinese sentences in 3 min silently and to evaluate whether each of them was literally correct or not.

#### 2.2.6. Working memory

The task measured the forward and backward digit span for assessing working memory (Richardson, 2007). During the assessment, digit sequences were presented to children, beginning with a length of two digits, which would increase every two trials, and the participants needed to recall the sequences by order or in reverse order.

Bilingual participants also completed a set of assessments of English literacy skills, including:

#### 2.2.7. English phonological awareness

The Elision subtest of the CTOPP (Wagner et al., 1999), which is similar to the Chinese phonological assessment.

#### 2.2.8. Morphological awareness

Early Lexical Morphology Measure (ELMM; Marks et al., 2021, see citation’s Appendix for the exact task). The paradigm was similar to the assessment of Chinese morphological awareness.

#### 2.2.9. Receptive vocabulary

Peabody Picture Vocabulary Test 5 (PPVT-5; Dunn, 2019)

Single-word reading: the Letter-word Identification subtest of Woodcock-Johnson IV (WJ-IV; Schrank et al., 2014), which is similar to the Chinese vocabulary assessment.

#### 2.2.10. Reading comprehension

The Passage Comprehension subtest of Woodcock-Johnson IV (WJ-IV; Schrank et al., 2014)

#### 2.2.11. Reading decoding skill

The Word Attack subtest of Woodcock-Johnson IV (WJ-IV; Schrank et al., 2014)

#### 2.2.12. English sentence reading fluency

The Reading Fluency subtest of Woodcock-Johnson IV (WJ-IV; Schrank et al., 2014), which is similar to the Chinese reading fluency test.

### 2.3. fNIRS stimuli and tasks

For the imaging task, a Chinese phonological task and an English phonological task were included, each comprising an experimental condition and a control condition. In the experimental condition, participants were auditorily presented with three spoken words sequentially, and were instructed to choose one of the last two words (i.e. the answer) that shared the same phonological onset with the first word (i.e. the target), but not the word that did not share the phonological onset with the first word (i.e. the distractor). All the three Chinese words in a single trial were two-character words, with the second character having the same sound among those words. The distractor was semantically related to the target, thereby requiring participants to focus solely on the phonological onset in order to identify the correct answer (e.g., three spoken words were 否則 (/foʊ_ˇ_tsɤˊ/, otherwise, the target) - 法則 (/faˇ tsɤˊ/, law, the answer) - 譴責 (/tɕʰjεňtsɤˊ/, condemn, the distractor). In the control condition during the Chinese phonological task, participants were also auditorily presented with three spoken words sequentially, with the same rule instructed. The answer words in this condition were identical to the targets, while the distractor words did not share the phonological onset with the targets (e.g., three spoken words were 新年 (/ɕɪnˉ njεnˊ/, new year, the target) - 新年 (/ɕɪnˉ njεnˊ/, new year, the answer) - 兔子 (/tʰuˋ tsɯ_˙_/, rabbit, the distractor). In both conditions, half of the answer words were presented before the distractors; and the other half were presented after the distractors.

The English phonological task followed the same rules as the Chinese phonological task, except that all the stimuli were English words. For example, the participants were auditorily presented with “Sunday (target) – subway (answer) – Monday (distractor)” in the experimental condition, and “hamster (target) – hamster (answer) – garlic (distractor)” in the control condition. The materials in the Chinese and English tasks were matched in terms of semantic relatedness between the target and the distractor in each trial (*p* = .07).

The Chinese task utilized stimuli that were digitally recorded by a female Chinese speaker, whereas stimuli for the English task were recorded by a female English speaker. Paired *t*-tests were conducted for both tasks to examine differences in word frequency between the experimental and control conditions. Word frequencies were assessed based on the survey report conducted by the National Languages Committee (2000) and the most recent version of the Corpus of Contemporary American English (COCA, Davies, 2020, https://www.english-corpora.org/coca/). For the Chinese task, the average word frequencies of the first characters were 1114.9 (*SD* = 1383.8) in the experimental condition, and 1511.0 (*SD* = 1783.7) in the control condition, respectively. The first characters were examined as they were the target of phonological segmentation. For the English task, the average word frequencies were 66,212 (*SD* = 114,556) in the experimental condition, and 84,716 (*SD* = 227,929) in the control condition, respectively. No significant difference between conditions was found in either task, indicating that word frequency was unlikely to determine the results.

In each trial, a 10-ms blank was present first, followed by a white fixation square displayed on the top of the screen for 1500 ms with the first auditory stimuli played. Then, a blue fixation square was displayed on the screen’s bottom-left for 1500 ms with the second auditory stimuli played. Finally, a yellow fixation square was displayed on the bottom-right of the screen for 3500 ms with the third auditory stimulus played. The participants were instructed to choose the answer between the left (i.e. the second) and the right (i.e. the third) words, using the keyboard on the laptop, as quickly and correctly as possible after they had heard the third stimuli. A blank interval of 1000 ms separated each trial, while a rest period of 3000 ms was presented after every four trials. Both tasks lasted approximately 7 min using E-prime 2 (Psychology Software Tools, Inc.) on a Lenovo G450 laptop. Auditory stimuli were presented through the laptop’s internal speaker at an approximated intensity of 75 db. Reaction times were calculated as the duration between the onset of the third stimulus and the button press for a response. Behavioral performance in the tasks was evaluated based on the accuracy and reaction times.

### 2.4. Experimental procedure

After the informed consent was signed, the head circumference of participants was measured, and the fNIRS cap and optodes were positioned. Then, the room lights were dimmed, and participants were given instructions. A practice session was conducted for both the Chinese and English tasks before the main experimental sessions. The practice session included both the experimental and control conditions, with practice stimuli designed to be distinct from those used in the testing sessions. Feedback was given after each practice trial to inform participants of the correctness of their choices, ensuring their understanding of the task rules. Then, the bilingual participants completed both the Chinese and English tasks in a counterbalanced order, and the monolingual participants completed only the Chinese phonological task.

### 2.5. Behavioral data analysis

The accuracy and reaction times of the bilingual group were analyzed by (1) the difference between the English and Chinese tasks with a 2 (task: Chinese, English) by 2 (condition: experimental, control) two-way ANOVA with two within-subject factors, to examine the performance of bilingual children across the two tasks. In addition, to compare the performance between bilingual and monolingual children in the Chinese task, the accuracy and reaction times were also analyzed with (2) the difference between bilingual and monolingual children doing the Chinese task by conducting a 2 (group: bilingual, monolingual) by 2 (condition: experimental, control) ANOVA with one between-subjects (group) and one within-subject (condition) factors.

### 2.6. fNIRS data recording

The hemodynamics response of participants was measured using 16 × 16 NIRScout (NIRx Medical Tech.), with illumination wavelengths set at 760 nm and 850 nm. A total of 16 near-infrared light emitters (sources) and 16 detectors were utilized, resulting in 39 data channels that were sampled at a rate of 3.91 Hz (Channels 1~19 in the right hemisphere, Channels 20~39 in the left hemisphere; Fig. 2a). These optodes were positioned on the fNIRS cap. According to previous fMRI studies, fNIRS neuroanatomical localization was set in the lateral frontal, parietal, and temporal regions for bilateral hemispheres, based on the 10–20 system, as regions of interest (Booth et al., 2002; Hickok and Poeppel, 2007; Lau et al., 2008). The cortical activity within these areas would increase during lexical tasks in Chinese or English (Booth et al., 2004; Brennan et al., 2013; Chou et al., 2006a, 2006b; Chou et al., 2009a, 2009b; Chou et al., 2019; Fan et al., 2010; Hickok and Poeppel, 2007; Hu et al., 2010; Lee et al., 2015, 2016; Mei et al., 2013, 2015; Müller et al., 2003; Zhu et al., 2014).

### 2.7. fNIRS data analysis

The fNIRS data were analyzed using the NIRS Brain AnalyszIR, a MATLAB-based analysis toolbox (Santosa et al., 2018). Given the necessity of a consistent number of trials for ensuring statistical power, all trials were included in the subsequent analyses, regardless of whether they were answered correctly (Bitan et al., 2007; Chen et al., 2016; Lai et al., 2021; Lee et al., 2011).

#### 2.7.1. Subject-level analysis

Raw data were first converted to hemoglobin concentration data, applying the modified Beer-Lambert law. Secondly, we trimmed the data collected from each channel by reserving 5 s before the onset of the first stimuli and 5 s after the last stimuli. Then, the trimmed hemoglobin concentration data were analyzed using the general linear model (GLM, Friston et al., 2007; Friston, 2009). The effects of confounding signals such as physiological or motion-induced signals were corrected using the AR-IRLS method (Barker et al., 2013). The signal drift over time was reduced by including temporal and dispersion derivatives, and also a DCT matrix with a cutoff frequency of 0.008 Hz in the model. The peak time of the hemodynamic response function (HRF) was set as 6 s (Brauer et al., 2008). This procedure yielded subject-level regression coefficients (beta values in each condition) for oxygenated hemoglobin (HbO) and deoxygenated hemoglobin (HbR) signals from each channel.

#### 2.7.2. Group-level analysis

Linear-mixed-effect (LME) models for each data channel were used to perform further group-level analysis. The first LME was fitted to examine the neural basis of phonological awareness during bilingual children performing the Chinese task and the English task. The task performed and the task conditions were modeled as a fixed effect, with participants as a random effect. The beta values of each subject for HbO and HbR as dependent variables. The corresponding formula was “beta ~ − 1 + Task:cond + (1|Subject)”, written in the Wilkinson Notation. The Estimated group-level effects of each channel were extracted to calculate the contrasts of experimental minus control conditions of (1.1) bilingual children during the Chinese task, (1.2) bilingual children during the English task, (1.3) the difference between the Chinese and English tasks in bilingual children.

The second LME was fitted to examine the neural basis of phonological awareness during bilingual and monolingual children performing the Chinese task. The participant group and the task conditions were modeled as fixed effects, with participants as random effects. The beta values of each subject for HbO and HbR as dependent variables. The corresponding formula was “beta ~ − 1 + Group:cond + (1|Subject)”, written in the Wilkinson Notation. The Estimated group-level effects of each channel were extracted to calculate the contrasts of experimental minus control conditions of (2.1) bilingual children during the Chinese task, (2.2) monolingual children during the Chinese task, (2.3) the difference between bilingual children and monolingual children during the Chinese task.

All the contrasts used only the HbO signal since HbO is the major contributor to the fNIRS signal (HbO~76 %; HbR~19 %; Strangman et al., 2002). The threshold level of significance in all the contrasts was set at *p* < .05, with family-wise error (FWE) correction for 39 channels.

To partial out task difficulty or proficiency, additional LME models were also fitted, while including behavioral performance (accuracy or reaction time or both) into the GLM model as covariate. The corresponding formula was listed below:

(1.a) “beta ~ − 1 + Task:cond + English_ACC + (1|Subject)”,

(1.b) “beta ~ − 1 + Task:cond + English_RT + (1|Subject)”,

(1.c) “beta ~ − 1 + Task:cond + English_ACC + English_RT + (1| Subject)”,

(2.a) “beta ~ − 1 + Group:cond + Chinese_ACC + (1|Subject)”,

(2.b) “beta ~ − 1 + Group:cond + Chinese_RT + (1|Subject)”, and

(2.c) “beta ~ − 1 + Group:cond + Chinese_ACC + Chinese_RT + (1| Subject)”, written in the Wilkinson Notation.

#### 2.7.3. Brain-behavior correlation

To further examine the relationship between the activities in brain regions and the children’s language skills, brain-behavior correlation analysis was conducted. The channels around the brain regions that showed significant activation in previous contrasts were set as regions of interest (ROIs). The beta values of the experimental minus control condition from ROI channels in each participant were extracted from the Subject-level GLM and imported into JASP (JASP Team, 2022).

We computed the correlation between the activation of the brain regions and behavioral assessments in Chinese and English. The Chinese CTOPP was used to evaluate phonological awareness in Chinese. The English PPVT, rather than English CTOPP, was chosen in English because English proficiency was relatively low, and vocabulary is closely related to phonological awareness in early development (Roth et al., 2002; Storch and Whitehurst, 2002). The threshold level of significance was set at *p* < .05, with family-wise error (FWE) correction for the number of significant channels in each model.

## 3. Results

### 3.1. Behavioral results

**Fig. 1.**
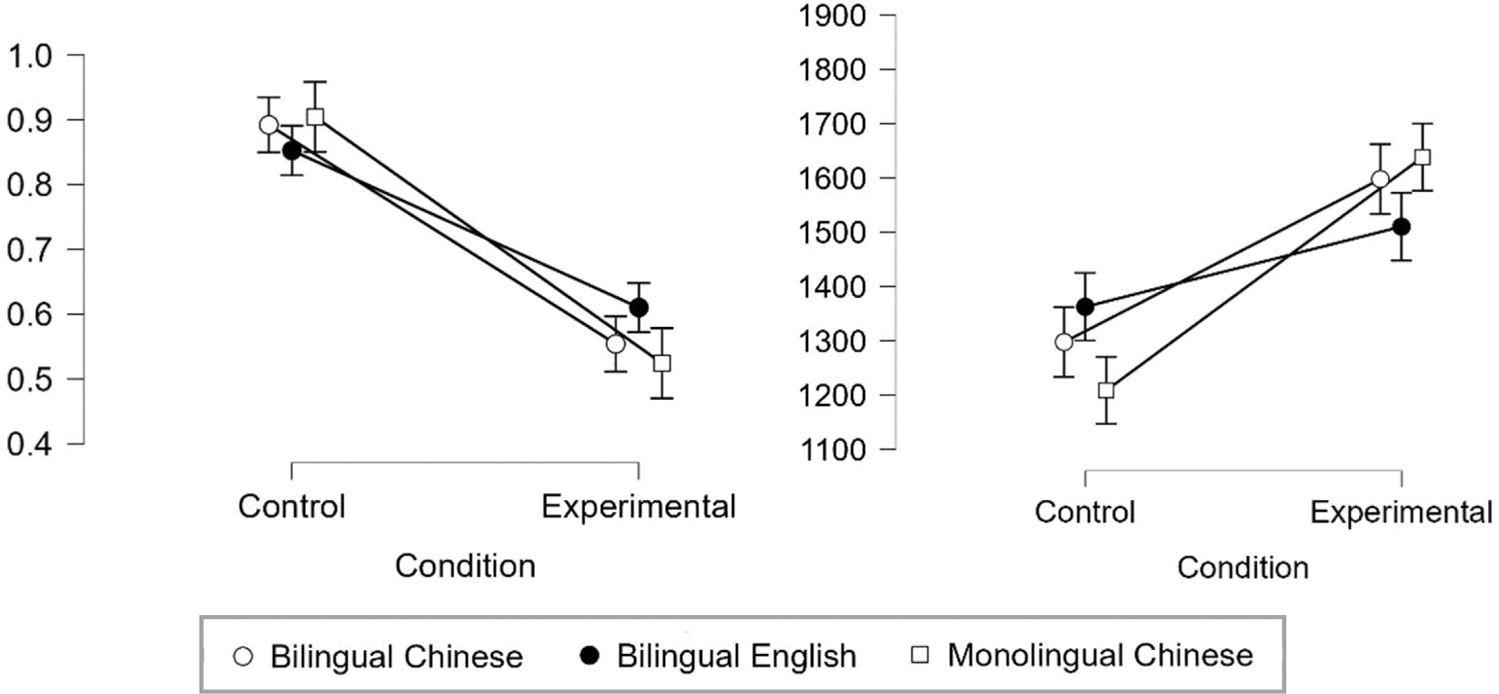
Neuroimaging Task Performance of Bilingual Children and Monolingual Children During the Chinese Task and English Task. Left: Accuracy; Right: Reaction Time.

For the literacy and linguistic tasks in Chinese, the difference between the bilingual and monolingual groups was nonsignificant (see Table 1). The behavioral performance of the fNIRS phonological tasks for monolingual and bilingual participants is presented in Fig. 1. We conducted two sets of data analysis (see Table 2).

#### 3.1.1. Difference between the english and chinese tasks in bilingual children

Regarding accuracy with a 2 (task: Chinese, English) by 2 (condition: experimental, control) ANOVA, the main effect of condition was significant. Post-hoc tests using Holm’s procedure showed significantly higher accuracy in the control condition (control-experimental: *t* = 13.550, *p* < .001), since the target and the answer were identical. The interaction was significant, and the simple main effect of task was significant in the experimental condition, with higher accuracy in the English task than in the Chinese task (*F* = 4.607, *p* = .040), but nonsignificant in the control condition (*F* = 2.905, *p* = .098).

Regarding reaction times, the main effect of condition was significant. Post-hoc tests using Holm’s procedure showed significantly faster reaction time in the control condition (experimental-control: *t* = 6.283, *p* < .001). The interaction was significant, and the simple main effect of task was significant in the experimental condition, with faster reaction times in the English task than in the Chinese task (*F* = 8.069, *p* = .008), but nonsignificant in the control condition (*F* = 2.010, *p* = .166).

#### 3.1.2. Difference between bilingual and monolingual children with the chinese task

Regarding accuracy with a 2 (group: bilingual, monolingual) by 2 (condition: experimental, control) ANOVA, the main effect of condition was significant. Post-hoc tests using Holm’s procedure showed significantly higher accuracy in the control condition (control-experimental: *t* = 15.254, *p* < .001).

Regarding reaction times, the main effect of condition was significant. Post-hoc tests using Holm’s procedure showed significantly faster reaction time in the control condition (experimental-control: *t* = 11.751, *p* < .001). The interaction was significant, but further analyses showed that the simple main effect of group was nonsignificant both in the control condition (*F* = 1.287, *p* = .261) and the experimental condition (*F* = 0.288, *p* = .593).

### 3.2. fNIRS results

#### 3.2.1. Bilingual children: Chinese vs. English

**Fig. 2.**
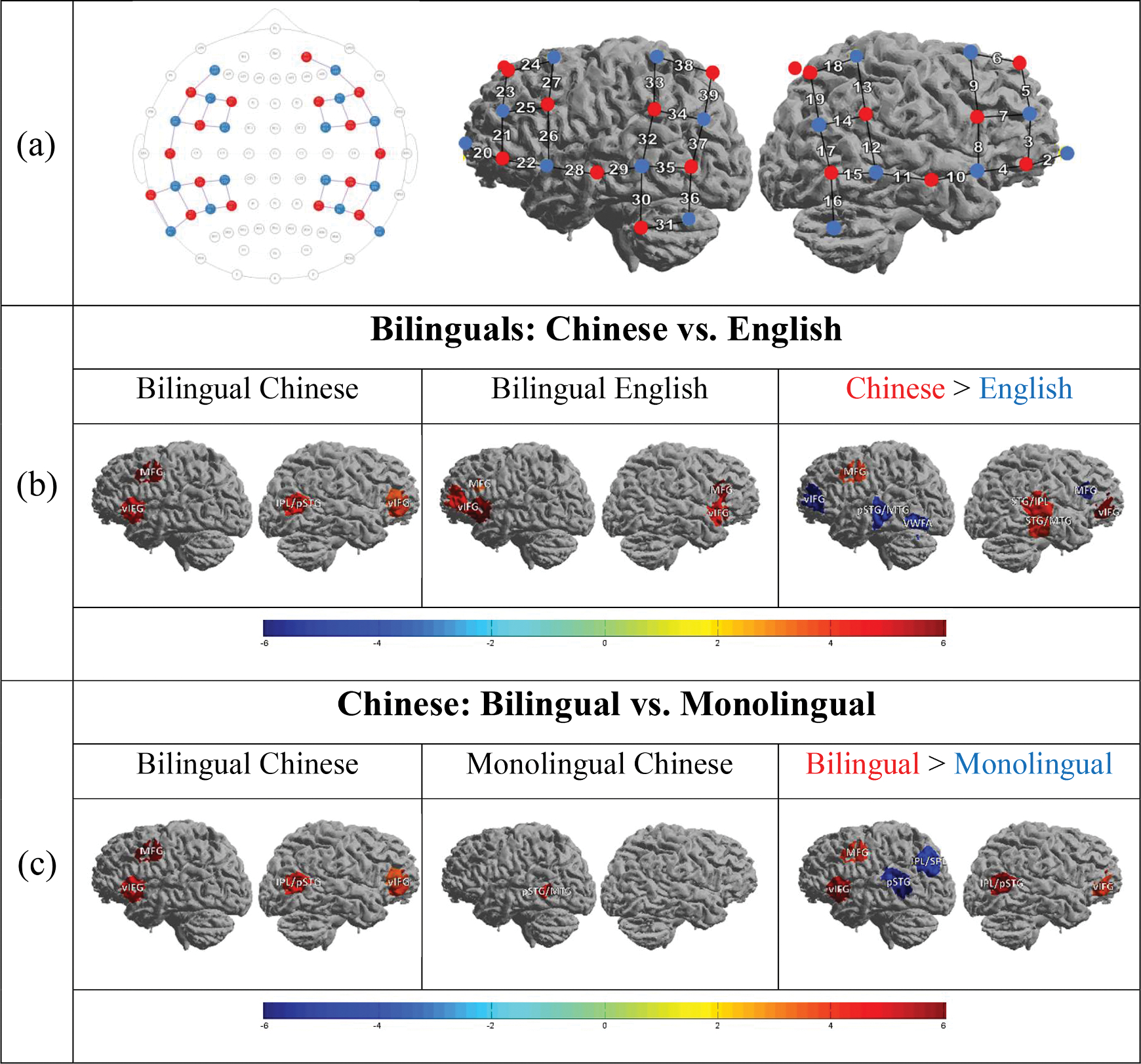
Brain activation of bilingual children with the Chinese and English task and monolingual children with the Chinese task. **(a)** fNIRS probe setup. Red dots: light sources; blue dots: light detectors; lines: data channels (Channel 1~19: right hemisphere; Channel 20~39: left hemisphere). **(b)** Comparison between brain activation of bilingual children with the Chinese and English task (*p*<.05). **(c)** Comparison between brain activation of bilingual and monolingual children with the Chinese task (*p*<.05).

In the Chinese task, bilingual children showed significant activation in 5 channels, over the bilateral vIFG/MFG and right IPL regions (see Table 3 and Fig. 2b). In the English task, bilingual children showed significant activation in 5 channels, over the bilateral vIFG/MFG regions (Fig. 2b). Direct comparison between two languages in bilinguals revealed that the Chinese task elicited stronger activation in 4 channels, mostly on the right hemisphere. In contrast, the English task elicited stronger activation in 4 channels, mostly over the typical reading network on the left hemisphere (IFG, pSTG/MTG; Fig. 2b). In sum, bilingual children showed bilateral frontal activation in both the Chinese task and the English task. However, brain regions on the right hemisphere were more involved when processing Chinese, and the typical reading network on the left hemisphere was more activated when processing English.

#### 3.2.2. Bilingual vs. monolingual children

During the Chinese task, bilingual children showed significant activation in 5 channels, over the bilateral vIFG/MFG and the right IPL regions (see Table 4 and Fig. 2c). Monolingual children showed significant activation in only 1 channel over the left pSTG/MTG (Fig. 2c). A direct comparison between the two groups revealed that bilingual children showed stronger activation in 4 channels, over the bilateral vIFG/MFG and the right IPL regions. In contrast, monolingual children showed stronger activation in 3 left channels, over the left pSTG/MTG and the left IPL/SPL regions (Fig. 2c). In sum, bilingual children showed more bilateral frontal activation when performing the Chinese task, while monolingual children showed significant activation only in the left temporal regions.

The significant effects listed above remain unchanged after controlling for accuracy or reaction time. In sum, our findings remain unchanged when we add task performance covariates to the analytical model.

#### 3.2.3. Brain-behavior correlation

**Fig. 3.**
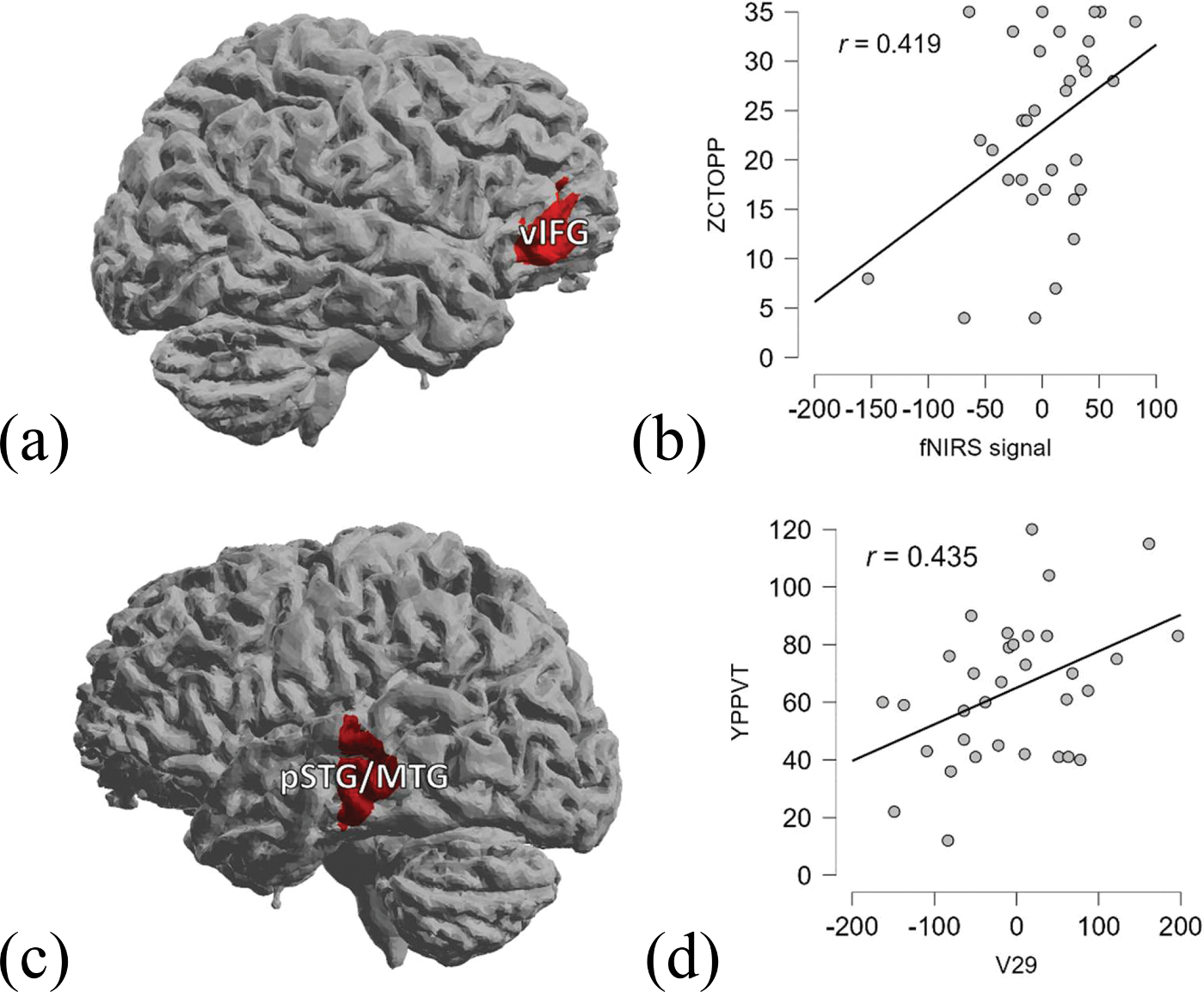
Brain-behavior correlation results **(a)** Significant brain-behavior correlation in the right vIFG **(b)** Distribution of the right vIFG activation in the Chinese task and raw scores of Chinese CTOPP in bilingual children. **(c)** Significant brain-behavior correlation in the left pSTG/MTG **(d)** Distribution of the left pSTG/MTG activation in the English task and raw scores of English PPVT in bilingual children.

The correlation between the beta values of the experimental condition minus the control condition and the scores of behavioral assessments was calculated. Based on the activated brain regions in the contrasts, for the bilinguals in the Chinese task, the channels that showed significant differences in activation during both the between-group comparison and the within-group comparison were selected as the ROIs for correlation analysis, including channels 2 (the right vIFG) and 27 (the left MFG). A positive correlation was found between the right mid-ventral IFG (channel 2) and the scores in the CTOPP (*Pearson’s r* = 0.419, *p* = .015; Fig. 3a, 3b).

For the bilinguals in the English task, since there was no between-group comparison, the channels that showed significant differences during the within-group comparison were selected as the ROIs for correlation analysis, including channels 7 (the right MFG), 20 (the left vIFG), 29 (the left pSTG/MTG), and 36 (the left VWFA). A positive correlation was found between the left pSTG/MTG (channel 29) and the scores in the English PPVT (*Pearson’s r* = 0.435, *p* = .011; Fig. 3c, 3d). For the monolingual group, the correlation analysis included the three channels that showed significant activation in the between-group contrast. However, no significant correlation was found in the monolingual group.

## 4. Discussion

The present study was conducted to examine the transfer effect in phonological processing between English and Chinese in first-grade students. We used an auditory phonological task that required phonological segmentation in both languages. Bilingual children in their early developmental stage were recruited for this study due to their heightened sensitivity to linguistic input during the ages of 6–8 (Werker and Hensch, 2015) and the significant correlation between phonological awareness development and literacy development in young Chinese-L1 children (Chow et al., 2005; McBride-Chang et al., 2005). In the present study, the within-subject comparison might be a reflection of language interaction for bilinguals, though the directionality of the transfer would be difficult to identify. Importantly, the between-subject comparison may reflect the L2-L1 transfer. Since both the bilingual and monolingual groups completed the Chinese (L1) task, the difference observed between these two groups would be the influence from English (L2) and thus the L2-L1 transfer during the Chinese task.

### 4.1. Bilingual children during the Chinese task and the English task

In the present study, after at least one year of English exposure, bilingual children in Taiwan performed better in the English task than in the Chinese task, which may show the early development of phonological awareness in English, in line with previous literature (Chen et al., 2010; Yeong and Rickard Liow, 2012). In terms of neural activation, the bilingual group showed activation in the bilateral frontal region in both the Chinese and English tasks. As compared to the Chinese task, left IFG activation was more widespread in English processing in bilingual children. Moreover, in the between-task contrast, the typical left language network (the left IFG, pSTG/MTG) was activated in the English task (Booth et al., 2002, 2004). On the other hand, as compared to the English task, more right-hemispheric regions (the right IFG and pSTG) were activated in the Chinese task. The results could be understood in the assimilation-accommodation framework (Nelson et al., 2008; Perfetti and Liu, 2005), that either using the procedures of the L1 network in the L2 acquisition (assimilation), or using L2 procedures that are different with L1 for reading the later acquired L2 (accommodation). Since the lateralization pattern of L2 English is different with that of L1 Chinese, the present study supports more on the accommodation effect in L2 English phonological processing for the young Chinese-English bilingual children.

The results may imply that even though English is the second language that is learned later than L1 (Chinese), bilingual children are more sensitive to English phonological information, and the typical left language network helps process phonology in English in the early-stage development. Such sensitivity could be associated with grapheme-phoneme analysis and the higher phonological transparency of the alphabetic language, which is in line with previous neuro-cognitive and developmental research (Brennan et al., 2013; Dehaene et al., 2015; Liu and Cao, 2016; McBride et al., 2021).

Among the typical language network, the importance of the left STG is highlighted for early literacy development in English (Brennan et al., 2013; Chyl et al., 2018; Martin et al., 2015). This region is a part of the dorsal neural pathway with phonological processes (Hickok and Poeppel, 2007), associated with automated phonological analyses such as manipulating units of sound (Ip et al., 2019). Previous studies have stated that higher phonological transparency may lead to greater involvement of the left temporal regions during language processing (Jamal et al., 2012; Paulesu et al., 2000). Since English is more phonologically transparent than Chinese, the significant correlation between left pSTG/MTG activation and English vocabulary by the PPVT also supports the importance of the temporal regions in early L2 English study.

### 4.2. Bilingual children and monolingual children during the chinese task

In the between-group comparison, both the behavior assessment and task performance were similar between the bilingual group and monolingual group, which may reflect a threshold of L2 proficiency. The L2-L1 transfer effect from English to Chinese, according to the literature, may be significantly observable after at least two years of English exposure (Chen et al., 2010), therefore further longitudinal results are needed. We found a marginally significant interaction with reaction time, suggesting a trend of faster responses by the bilingual group relative to the monolingual group during the Chinese task. Yet, the simple main effect was not significant and thus, a longitudinal study is likely needed to elucidate this result further.

In terms of neural activation, monolingual children showed activation in the left STG and IPL. These regions were reported as the dorsal neural pathways with phonological analysis in alphabetic languages (Bolger et al., 2005; Booth et al., 2004; Brennan et al., 2013), and their role in Chinese character processing was highlighted in language tasks with higher demands on phonological processing (Feng et al., 2020; Hu et al., 2010; Li et al., 2022).

On the other hand, in spite of the behavioral inefficient outcome, bilingual children showed greater activation of the bilateral IFG, which may demonstrate more manipulation mechanisms involved when performing the task (Wang et al., 2021). Given that the frontal lobe matures later than the temporal lobe in children (Skeide and Friederici, 2016), previous studies have found that the IFG did not show phonological specialization until age 7–8, suggesting the development in phonemic manipulation after 1-to-2 years of formal education in the US (Wang et al., 2021; Weiss et al., 2018). In the present study, compared to the absence of IFG activation in monolingual children, the bilateral IFG activation in bilingual children suggests early frontal involvements in phonological processing. Since bilingual children have early exposure to English, a more phonologically transparent language, greater activation in the IFG may indicate a transfer effect from L2 to L1 on the early development of phonological manipulation, which would lead to higher phonological awareness (Kilpatrick, 2015).

Interestingly, in addition to the left IFG, the right IFG was activated in bilingual children. Right IFG activation was also shown in the between-task comparison, which may possibly be explained by the tonal information (Wong, 2002; Yu et al., 2014) or the visuo-spatial feature of logographic language (Gazzaniga et al., 1965; Wu et al., 2012). However, since such activation was also stronger when compared to the monolingual children, it could not be explained solely by the characteristics of Chinese processing. One possible explanation is that right IFG activation may be related to the compensation of the left IFG in the analytic demands with the phonological opaqueness of Chinese. This may lead to higher activation in bilingual children to deal with phonological segmentation in Chinese.

Based on the brain-behavior correlation analyses in the present study, the positive correlation between right IFG activation and phonological awareness suggests that the involvement of the right IFG may play a pivotal role in the development of phonological ability in early Chinese-English bilingual children. Prior research has revealed that right IFG activation can support lexical processing through additional processing to facilitate lexical retrieval/selection in the left hemisphere (Riès et al., 2016). Therefore, the right IFG activation could be seen as facilitation to support the learning of phonological ability. Also, the neuroimaging findings in the present study may indicate that with the limited bilingual exposure, a transfer effect from L2 could be found, starting with the bilateral IFG involvement to incorporate dual-language information to support phonological processing actively.

### 4.3. Limitations

The current study has limitations. First, the study’s ability to explore a strong bilingual effect is restricted due to the relatively small number of monolingual participants. Second, the English task was not examined in monolingual groups due to time constraints, thus limiting the possible evidence of L1-L2 transfer effect on English language acquisition. However, extensive literature has already discussed this aspect. Third, given that the participants were in the early stages of Zhuyin acquisition, their phonological awareness in Chinese may have been insufficient to complete the Chinese phonological task with high accuracy, potentially contributing to a trend of behavioral effects between the two groups.

### 4.4. Conclusion

In conclusion, this study aimed to investigate the behavioral and neural processes involved during phonological processing in young Chinese-English bilingual children, especially with the potential transfer effect. The bilingual children showed greater activation mainly in the left dorsal neural pathway (left IFG, pSTG/pMTG) in English, showing higher sensitivity to English phonological information, and the importance of the left temporal regions is highlighted in the sensitivity. In Chinese phonological processing, bilingual children showed greater involvement of the bilateral IFG than monolingual children, demonstrating more analytic mechanisms and increased sensitivity to the dual language information, which may indicate further transfer effect. These results elucidate the developmental adjustments in the neural substrates associated with early bilingual exposure in phonological processing, offering valuable insights regarding the benefits and drawbacks of early second language acquisition.

## Figures and Tables

**Table 1:** Behavioral Performance in Linguistic Tasks in Raw Scores (Means ± SDs) and fNIRS Phonological Task.

	Monolingual (*N* = 29)	Bilingual (*N* = 33)	*t*	*P*

***Chinese*** (score range)				
Digit Span	15.5 ± 3.65	14.4 ± 2.76	−1.376	0.174
Chinese PPVT	79.1 ± 11.45 (62–102)	78.0 ± 11.01 (56–98)	−0.409	0.684
Morphological construction	15.1 ± 4.92 (6–25)	14.8 ± 5.09 (4–24)	−0.200	0.842
Character Recognition	21.8 ± 6.83 (7–30)	19.7 ± 6.97 (8–30)	−1.176	0.244
Character Reading	24.6 ± 17.62 (0–54)	22.1 ± 16.81 (1–53)	−0.578	0.565
CTOPP Elision	23.3 ± 9.69 (7–36)	23.1 ± 9.31 (4–35)	−0.103	0.918
Reading Fluency	13.3 ± 12.56 (0–40)	10.8 ± 12.61 (0–37)	−0.789	0.433
***English*** (score range)				
Extract the Base		4.5 ± 5.63		
PPVT		64.3 ± 24.69 (12–120)		
CTOPP Elision		14.1 ± 6.24 (0–27)		
Letter Word Identification		26.3 ± 8.68 (11–46)		
Passage Comprehension		12.6 ± 4.08 (4–20)		
Word attack		11.7 ± 3.75 (4–19)		
Reading Fluency		8.6 ± 7.73 (0–33)		

*Note*. English PPVT: Peabody Picture Vocabulary Test 5 (PPVT-5, Dunn, 2019); Chinese PPVT: Peabody Picture Vocabulary Test-Revised (PPVT-R, Lu and Liu, 1998); English CTOPP: Elision subtest of the Comprehensive Test of Phonological Processing, (CTOPP, Wagner et al., 1999); Chinese CTOPP: a Chinese version of the Elision subset of English CTOPP based on Newman et al. (2011).

**Table 2:** fNIRS phonological task accuracy and reaction time ANOVA analysis results.

Bilinguals: Chinese vs. English									

*Accuracy*	*F*	*df*	*p*	$\eta_{G}^{2}$	*Reaction Time*	*F*	*df*	*p*	$\eta_{G}^{2}$

Task	0.241	32	.627	< .001	Task	0.146	32	.705	< .001
Condition	183.609	32	< .001	.697	Condition	39.477	32	< .001	. 345
Task*Condition	7.051	32	.012	.019	Task*Condition	8.817	32	.006	.040
Chinese: Bilingual vs. Monolingual									
*Accuracy*	*F*	*df*	*p*	$\eta_{G}^{2}$	*Reaction Time*	*F*	*df*	*p*	$\eta_{G}^{2}$
Task	0.074	60	.786	< .001	Task	0.119	60	.732	< .001
Condition	232.696	60	< .001	.575	Condition	138.089	60	< .001	.270
Task*Condition	0.801	60	.374	.002	Task*Condition	4.345	60	.041	.008

**Table 3:** Brain activation of bilingual children with the Chinese and English task.

Hemisphere	Channel	Region	beta	*t*-stat	*p (FWE)*

**Bilingual Chinese**					
R	2	vIFG	15.39	3.97	.0031
R	3	MFG/vIFG	12.24	3.58	.0143
R	17	IPL/pSTG	18.67	4.35	.0006
L	22	vIFG	21.49	4.75	.0001
L	27	MFG	32.81	6.22	<.0001
**Bilingual English**					
R	4	vIFG	24.07	4.36	.0006
R	7	MFG	24.38	5.74	<.0001
L	20	vIFG	18.21	4.43	.0004
L	22	vIFG	32.09	6.21	<.0001
L	25	MFG	17.30	3.55	.0161
**Bilingual Chinese > Bilingual English**					
R	2	vIFG	27.80	5.25	<.0001
R	11	STG/MTG	22.90	3.57	.0153
R	12	STG/IPL	27.90	3.71	.0090
L	27	MFG	24.67	3.29	.0413
**Bilingual English > Bilingual Chinese**					
R	7	MFG	−28.39	−5.03	<.0001
L	20	vIFG	−28.83	−4.97	<.0001
L	29	pSTG/MTG	−31.23	−3.81	.0060
L	36	VWFA	−43.00	−3.64	.0114

Note: vIFG: ventral inferior frontal gyrus; MFG: middle frontal gyrus; STG: superior temporal gyrus; pSTG: posterior superior temporal gyrus; MTG: middle temporal gyrus; IPL: inferior parietal lobe; VWFA: visual word form area.

**Table 4:** Brain activation of bilingual and monolingual children with the Chinese task.

Hemisphere	Channel	Region	beta	*t*-stat	*P (FWE)*

**Bilingual Chinese**					
R	2	vIFG	15.66	3.94	.0036
R	3	MFG/vIFG	12.34	3.52	.0183
R	17	IPL/pSTG	19.12	4.34	.0007
L	22	vIFG	21.27	4.58	.0002
L	27	MFG	32.72	6.04	<.0001
**Monolingual Chinese**					
L	35	pSTG/MTG	16.68	4.70	.0002
**Bilingual > Monolingual**					
R	2	vIFG	18.85	3.57	.0150
R	17	IPL/pSTG	27.82	5.14	<.0001
L	22	vIFG	31.87	4.97	<.0001
L	27	MFG	30.77	3.72	.0085
**Monolingual > Bilingual**					
L	32	pSTG	−27.41	−4.11	.0018
L	35	pSTG/MTG	−33.71	−5.51	<.0001
L	39	IPL/SPL	−27.38	−4.02	.0026

Note: vIFG: ventral inferior frontal gyrus; MFG: middle frontal gyrus; STG: superior temporal gyrus; pSTG: posterior superior temporal gyrus; MTG: middle temporal gyrus; IPL: inferior parietal lobe; SPL: superior parietal lobe.

## Data Availability

Inquiries of data and code can be directed to the corresponding author by consulting with the local ethics committee.
